# Four Decades of COPD Mortality Trends: Analysis of Trends and Multiple Causes of Death

**DOI:** 10.3390/jcm10051117

**Published:** 2021-03-07

**Authors:** Alberto Fernández-García, Mónica Pérez-Ríos, Alberto Fernández-Villar, Gael Naveira, Cristina Candal-Pedreira, María Isolina Santiago-Pérez, Cristina Represas-Represas, Alberto Malvar-Pintos, Sara Cerdeira-Caramés, Alberto Ruano-Raviña

**Affiliations:** 1 Department of Preventive Medicine and Public Health, School of Medicine, University of Santiago de Compostela, 15772 Santiago de Compostela, Spain; alberto.fernandez.garcia@outlook.es (A.F.-G.); cristina.candal.pedreira@rai.usc.es (C.C.-P.); mariaisolina.santiago.perez@sergas.es (M.I.S.-P.); alberto.ruano@usc.es (A.R.-R.); 2 Department of Radiology, Hospital POVISA, 36211 Vigo, Spain; 3 Consortium for Biomedical Research in Epidemiology and Public Health (CIBER en Epidemiología y Salud Pública, CIBERESP), 28029 Madrid, Spain; 4 C013 Group-Health Research Institute of Santiago de Compostela (Instituto Investigación Sanitaria de Santiago de Compostela/IDIS), 15706 Santiago de Compostela, Spain; 5 Pulmonology Department, Alvaro Cunqueiro University Hospital, 36312 Vigo, Spain; alberto.fernandez.villar@sergas.es (A.F.-V.); cristina.represas.represas@sergas.es (C.R.-R.); 6 NeumoVigo I+I Research Group, Institute of Health Research Galicia Sur, 36612 Vigo, Spain; 7 Epidemiology Unit, Galician Health Authority, Xunta de Galicia, 15701 Santiago de Compostela, Spain; estatistica.SP@sergas.es (G.N.); alberto.malvar.pintos@sergas.es (A.M.-P.); sara.cerdeira.carames@sergas.es (S.C.-C.)

**Keywords:** COPD, trends, mortality, multiple causes

## Abstract

There is little information on chronic obstructive pulmonary disease (COPD) mortality trends, age of death, or male:female ratio. This study therefore sought to analyze time trends in mortality with COPD recorded as the underlying cause of death from 1980 through 2017, and with COPD recorded other than as the underlying cause of death. We conducted an analysis of COPD deaths in Galicia (Spain) from 1980 through 2017, including those in which COPD was recorded other than as the underlying cause of death from 2015 through 2017. We calculated the crude and standardized rates, and analyzed mortality trends using joinpoint regression models. There were 43,234 COPD deaths, with a male:female ratio of 2.4. Median age of death was 82 years. A change point in the mortality trend was detected in 1996 with a significant decrease across the sexes, reflected by an annual percentage change of −3.8%. Taking deaths into account in which COPD participated or contributed without being the underlying cause led to an overall 42% increase in the mortality burden. The most frequent causes of death when COPD was not considered to be the underlying cause were bronchopulmonary neoplasms and cardiovascular diseases. COPD mortality has decreased steadily across the sexes in Galicia since 1996, and age of death has also gradually increased. Multiple-cause death analysis may help prevent the underestimation of COPD mortality.

## 1. Introduction

Chronic obstructive pulmonary disease (COPD) is currently one of the most common diseases, and its population impact is expected to increase over the coming years [1,2]. Although COPD prevalence varies widely among countries, in Spain, the cross-sectional study EPISCAN II aimed at estimating COPD prevalence showed that it was 12% among adults over the age of 40 years [3]. These data are very similar to those reported by the Global Burden of Disease, which estimated a global prevalence of 11.7% [4]. Furthermore, COPD gives rise to high mortality, estimated at 3.17 million deaths worldwide in 2015, accounting for 5% of total mortality [4]. Available studies indicate that there has been a decline in COPD mortality since the 1990s, especially among men [5,6], though it is not known exactly when this decrease began because there are no long-term time series data [7]. Hence, having mortality data based on a large population study conducted in a single area sharing the same healthcare model would yield valuable information.

Mortality rates due to any cause can be expressed in various ways, depending upon the objectives to be assessed. The crude mortality rate expresses the relationship between the number of deaths occurring in any given period and the size of the population among whom these occur, and is of clinical utility since it allows for local evaluation [8]. The standardized (or adjusted) mortality rate expresses this relationship, while avoiding the influence of differences in the structure of the populations analyzed by, for instance, age, thereby rendering it possible to make comparisons between rates derived from populations with different age structures (e.g., comparisons between the rates of different countries or different moments in time) [5,6].

Since the International Classification of Diseases (ICD) was introduced, causes of death are, on the whole, uniformly registered and are analyzed by the selection of a single cause, known as the underlying cause [7]. However, it should be noted that, as indicated by the GOLD (Global Initiative for Chronic Obstructive Lung Disease) guide [2], COPD mortality data are likely underestimated for several reasons. On the one hand, there is a high rate of underdiagnosis of the disease; on the other, COPD is not included as the cause of death in many instances where the death is coded as due to another cause, without considering that the primary cause might indeed be COPD [9,10,11,12]. The progressive increase in the mean age of patients who die of COPD in Spain only serves to further complicate the problem of accuracy in the coding of cause of death [12,13]. It is often difficult to establish whether a patient has died “due to” or “with” COPD, in view of this disease’s frequent coexistence with other cardiovascular comorbidities or neoplasms [13].

The section of the Spanish death statistics report form (Boletín Estadístico de Defunción) addressing health status, as opposed to information of a purely demographic nature, lists four steps in the dying process (immediate, intermediate, initial or fundamental, and other processes), which must be completed. If the certification is performed properly, the cause shown as initial or fundamental is then chosen as the underlying cause of death, since it is this disease that is regarded as having initiated the chain of events which culminated in death [7]. Among patients with COPD, it is essential to ascertain precisely how their deaths are being certified because this could provide us with a more comprehensive overview of the problem. To date, this aspect has hardly been studied and could be of enormous utility for other studies that seek to analyze COPD mortality in Spain and other countries, since the choice of cause of death meets an international standard.

Accordingly, the aims of this study were to analyze, firstly, the time trend in mortality due to COPD considered as the underlying cause of death, across a period of almost 40 years (longer than any previous study in Spain), and secondly, the registration of COPD along with other possible causes of mortality not considered as the underlying cause of death.

## 2. Methods

### 2.1. Data Sources

We performed an analysis of deaths due to COPD among persons aged 35 years and over in Galicia (NW Spain) across the period 1980–2017, using data sourced from the Galician Regional Deaths Register. For study purposes, we included both deaths with COPD shown as the underlying cause across the entire period (mortality “due to COPD”), and deaths with COPD shown as the immediate cause, intermediate cause, or other process (mortality “with COPD”) for the years 2015 through 2017. In all cases, COPD was identified by ICD-9 codes 491, 492, and 496 across the period 1980–1998, and ICD-10 codes J41–44 across the period 1999–2017. In addition, we recorded the main ICD-10 diagnostic codes of deceased patients, among whom COPD was shown as the immediate cause, intermediate cause, or other process.

### 2.2. Statistical Analysis

(a) Assessment of mortality with COPD as underlying cause of death.

For each year across the period 1980–2017, we calculated COPD-specific mortality rates by five-year age group, and crude rates and age-standardized rates in the population aged 35 years and over, by sex.

For the calculation of rates, annual population data for the years 1980–1997 were drawn from intercensal estimates calculated on the basis of the 1981 and 1991 population censuses and the 1986, 1996, and 1998 electoral rolls. For the period beginning in 1998, data were obtained from the Annual Electoral Roll Update, as sourced from the Galician Statistics Institute. Standardization rate was performed with the direct method using the 2011 Galician census population as standard.

(b) Analysis of multiple causes of mortality due to COPD as a contributing cause of death.

Across the period 2015–2017, we calculated annual crude mortality rates due to COPD in cases where COPD was included as the underlying cause, and annual crude mortality rates with COPD in cases where COPD was included as a contributing cause (immediate, intermediate, or other process) without being shown as the underlying cause on the death certificate.

(c) Analysis of trends.

To ascertain the time trend in the annual standardized rates, joinpoint regression models were fitted on the basis of logarithmic transformation of the mortality rates. For each of the adjusted segments that enable identification of the cut-off points at which statistically significant changes in the trend take place, we calculated the annual percentage change (APC) along with its 95% confidence interval (95% CI) [10].

All rates are expressed as cases per 100,000 inhabitants. Data were analyzed with the Stata 14 software program, and the joinpoint regression was performed using the Joinpoint Regression software program v4.0.4 (National Cancer Institute, USA) [14,15].

## 3. Results

### 3.1. COPD Mortality Trend: Period 1980–2017

Across the period 1980–2017, there were 43,234 deaths due to COPD among the population aged 35 years and over in Galicia. Of this total, 30,383 deaths occurred in men, with a male (male:female) mortality ratio (MR) of 2.4. The lowest number of deaths was observed in 1980, with 597 deaths, and the highest number in 1996, with 1502 deaths. In 2017, there were 1031 deaths due to COPD in Galicia, with an MR of 2.5. Broken down by age group, the MR was highest in the groups aged 35 through 39 years and 55 through 59 years—that is, for every woman that died, 5.5 men died. The median age of death was 82 years, with this being higher in women than in men (85 vs. 81 years) (Table 1).

**Figure 1:**
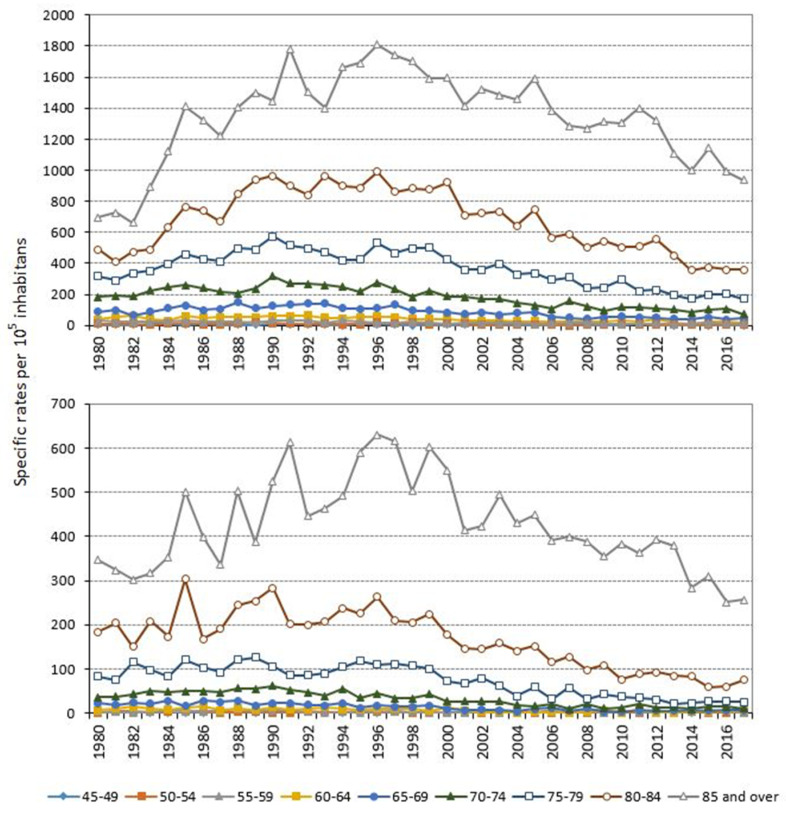
Annual specific mortality rates due to chronic obstructive pulmonary disease (COPD) as underlying cause of death in men and women. Galicia, population aged 45 years and over, period 1980–2017.

In all the years across the period, COPD-specific mortality rates were virtually zero up to age 45 years, and from age 45 upwards, rose with age in men and women alike (Figure 1). COPD mortality peaked in persons over the age of 84 years, with an MR of 1.4.

**Figure 2:**
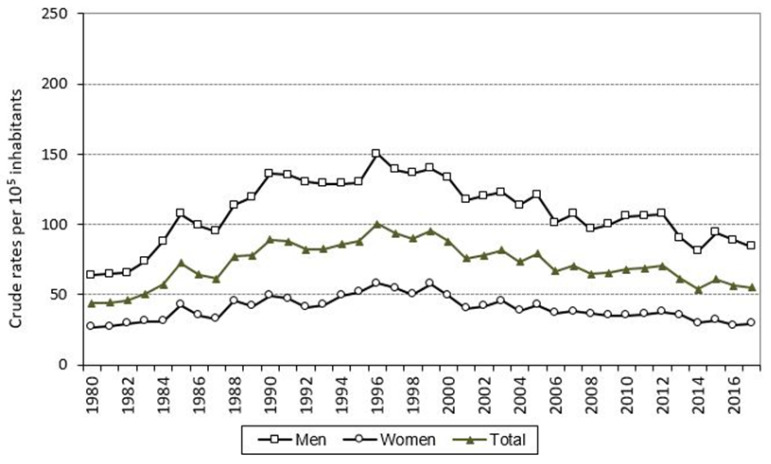
Annual crude mortality rates due to COPD as underlying cause of death, both overall and in men and women. Galicia, population aged 35 years and over, period 1980–2017.

**Figure 3:**
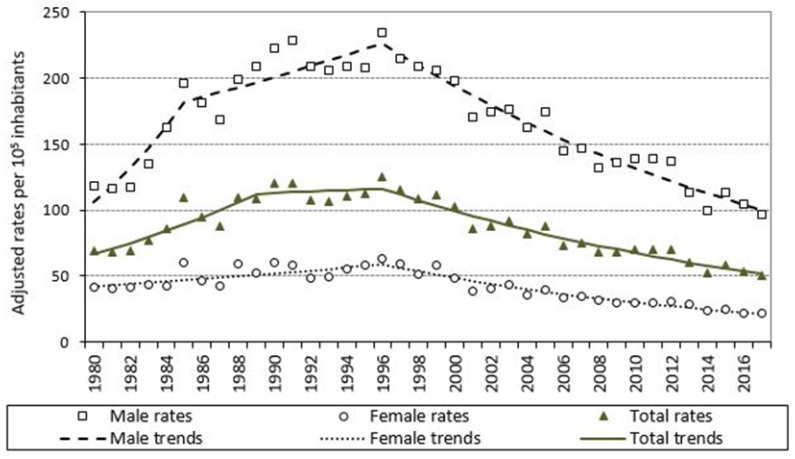
Standardized mortality rate due to COPD as underlying cause of death, both overall and in men and women, and trend in standardized rates. Galicia, population aged 35 years and over, period 1980–2017.

**Figure 4:**
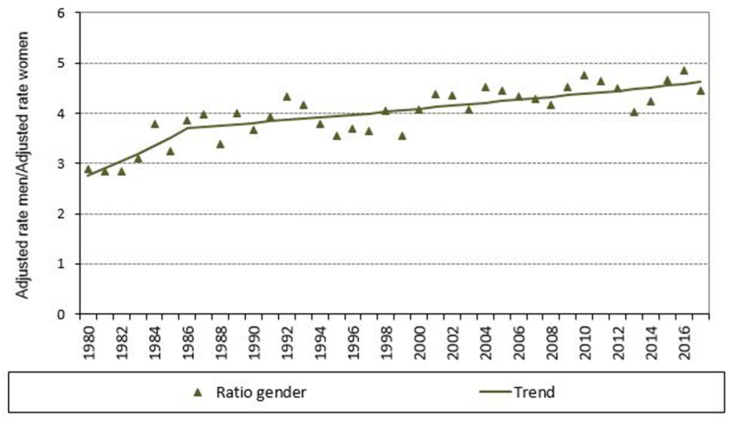
Ratio of age-standardized mortality rates due to COPD as underlying cause of death, both overall and in men and women, and trend in age-standardized ratios. Galicia, population aged 35 years and over, period 1980–2017.

The trend in the crude COPD mortality rate in the population aged 35 years and over is shown in Figure 2, both overall and by sex. The mortality rate increased in both sexes until 1996 and then decreased until 2017, which was when these crude rates, like the standardized rates (Figure 3), registered the lowest values in the series. The age-standardized mortality ratio increased during the period, reaching the highest value in 2017 (Figure 4). When the time trends were analyzed in detail (Figure 3), a change point in the COPD mortality trend was detected in 1996, after which there was a significant decrease in mortality, reflected in an APC of −3.8% (95% CI: −4.3 to −3.3). This significant decrease was observed in both men and women (Table 2).

### 3.2. Trend in Mortality Due to COPD and with COPD: Period 2015–2017

Across the period 2015–2017, COPD was considered to be the underlying cause in 3227 deaths. In contrast, COPD was considered not to have been the underlying cause but to have participated in the dying process as the immediate or intermediate cause in 1013 deaths, and as the contributing cause—included under “Other processes”—in 1331 deaths (Table 3). Deaths in which COPD participated or contributed to the death process accounted for 42% of the total burden of mortality due to COPD.

**Figure 5:**
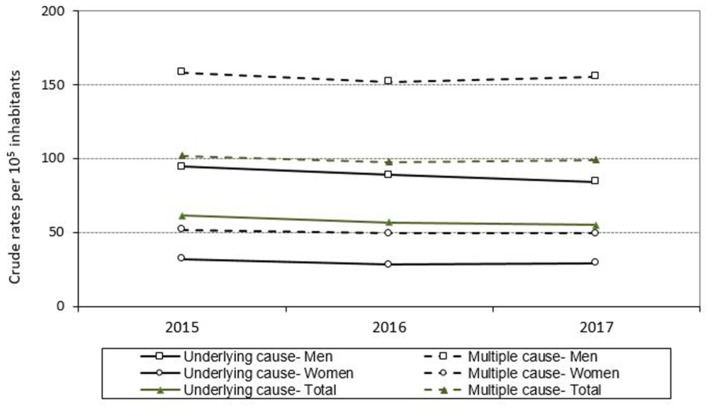
Crude mortality rates “due to” COPD (COPD as underlying cause: solid line in the figure) and “with” COPD (COPD as underlying cause and as immediate, intermediate, or other process (multiple causes: dotted line in the figure)) on the death certificate, both overall and by sex. Galicia, population aged 35 years and over, period 2015–2017.

In terms of the trend in the crude mortality rates across these 3 years (Figure 5), when only the underlying cause of death was considered, a decrease of 10.2 percentage points was observed in the male mortality rate and 2.5 percentage points in the female mortality rate (6.1 percentage points overall). On the other hand, when the crude mortality rates considered COPD included in any cause of death, the differences were 2.9 percentage points in men and 2.5 percentage points in women (2.6 overall).

**Figure 6:**
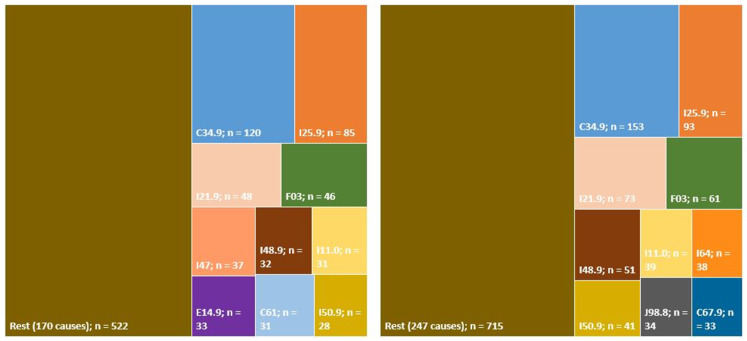
Diagram proportionally depicting ICD-10 codes considered as the underlying cause of death, in which COPD is recorded as immediate or intermediate (**left**), or as other process (**right**): period 2015–2017. ICD-10 codes included (order of frequency): C34.9: malignant neoplasm of bronchus or lung; I25.9: chronic ischemic heart disease; I21.9: acute myocardial infarction; F03: dementia, unspecified; I48.9: atrial fibrillation; I47: paroxysmal tachycardia; I50.9: heart failure, unspecified; I11.0: hypertensive heart disease; I64: stroke; J98.8: other respiratory disorders; E14.9: diabetes mellitus, unspecified; C67.9: cerebrovascular disease; and C61: malignant neoplasm of prostate. “Rest” includes 170 causes in the figure at left and 247 causes in the figure at right; “n” refers to the number of deaths included in each category.

Over half of the causes shown as the underlying cause of death when COPD appeared as immediate, intermediate, or other processes were linked to 10 causes of mortality. The most frequent ICD-10 codes considered as the underlying cause of death in cases where COPD was shown as immediate, intermediate, or other processes, but not as the underlying cause, are depicted in Figure 6. The four most frequent causes coincided in cases where COPD appeared as an immediate or intermediate cause, but not an underlying cause, and where COPD appeared under “Other processes”. These were malignant neoplasm of unspecified part of bronchus or lung (C34.9), ischemic heart disease (I25.9), acute myocardial infarction (I21.9), and unspecified dementia (F03). In the next six causes, the following three were repeated: hypertensive heart disease (I11.0); atrial fibrillation (I48.9); and heart failure, unspecified (I50.9) (Figure 6).

## 4. Discussion

This study furnishes novel information on trends in COPD mortality and the impact this can have on the certification of multiple-cause mortality on the medical death certificate. In 1996, there was a change in the mortality trend, with a continuous annual decrease of almost 4% until 2017. When it comes to multiple causes of death, COPD is frequently included in other causes of death that are not considered to be the underlying cause, something that may serve to underestimate the true current influence of this disease on mortality statistics, and could have implications in any future COPD mortality studies conducted using multiple-mortality coding.

This study represents the longest updated COPD mortality time series analyzed in Spain and Europe [5,6,7]. There is only one study, undertaken by Lopez-Campos et al. in Andalusia, which includes data since 1975, but it ends in 2010 [7]. National data included in other international studies only date from 1990 onwards [5,6]. From 1980, the rise in COPD mortality in Galicia is seen to gradually level off until 1995 (before reaching the inflection point in 1996)—a trend that is more clearly visible among men. Both trends are evident in men and women, and display no differences by sex. These data are in line with existing literature. Another study by Lopez-Campos et al. analyzed the mortality trends of 27 European countries from 1994 through 2000, reporting a downward trend in both sexes. While the mortality rate in men fell from 90.1 cases per 100,000 inhabitants in 1994 to 61.3 in 2010, the decrease was less marked in women, going from 27.0 to 25.2 [5]. For Spain, this study indicates that the COPD mortality rate decreased across the study period in men and women alike. The study conducted by the same author in Andalusia reports similar results, with a downward trend in the period 1994–2010, in men (from 149.1 to 98.0 cases per 100,000 inhabitants) and women (from 26.4 to 12.0) [7]. However, the trend in the first two decades in Andalusia shows a bimodal behavior pattern, with a clear decrease from 1975 through 1981, and an increase until 1993 in women and 1997 in men, which is when the downward trends begin. Similar to our study, the data from Andalusia show a more pronounced rise in the first half of the 1980s and a more gradual rise until the mid-1990s, marking the start of the clear downward trend [7].

Many reasons account for this change in trend from the mid-1990s onwards. From a clinical standpoint, mention should be made of the increasingly generalized use of long-acting bronchodilators in these patients, and of non-invasive mechanical ventilation in patients with hypercapnic respiratory failure, as well as enhanced knowledge of the disease, its control, and the management of its comorbidities [7,16,17]. Another possible explanation might lie in the change in ICD codes, following the switch from the ninth to the tenth revision that took place during this decade. Even so, there are studies which have shown that this is not the cause of under-registration of COPD mortality [18,19], something that goes to reinforce both the findings of this and other similar studies, and the reasons for it explained above.

In addition to advances in the care of the disease, the measures being implemented in the majority of the most developed countries could positively influence the trends described in this and other studies. These measures focus on targeting smoking habit, environmental pollution and occupational exposures to different types of smoke and other toxics, reducing underdiagnosis of the disease, and controlling the different respiratory and non-respiratory comorbidities [20]. Different authors have estimated that this downward trend may become markedly steeper over the following two decades, so that by or around 2040, most affected patients may die “with” rather than “due to” COPD [5,21]. This is further reinforced by the median age of death—across the period 2010 through 2017, the median age of death was 81 years in men and 85 in women, being equal to the life expectancy of men and slightly lower than that of women (National Statistics Institute/Instituto National de Statistical (INE)).

This finding heightens the relevance of analyzing the completion of death certificates. In this study, just over 40% of death certificates included COPD as part of the dying process, implying that the real burden of the disease in the mortality statistics is considerably higher. In clinical hospital cohort studies published in Spain, the most frequent causes of mortality reported in patients with COPD are neoplasms and cardiovascular diseases [22,23]. This is in line with our study in that when COPD is not considered to be the underlying cause of death, most of the patients die due to neoplasms (essentially bronchopulmonary), different cardiovascular diseases, or dementias. Although this finding has already been observed in studies undertaken in other countries [11,12], it is evident that analysis of mortality data, if not completed with multiple causes, does not reflect the real burden of COPD at a population level. This is an increasingly important problem in COPD, both for the reasons discussed and the progressive increase in the age of death, which in our study was around two years per decade. It is therefore essential to have all data shown on the certificates (multiple causes of death), and to analyze these in order to better understand how the different diseases making up the current mortality scenario behave [8,12]. Although this multiple coding was available to us for a period of only 3 years and the underlying cause-based mortality trend is also dropping (particularly in men), when we used multiple-cause coding, the drop nonetheless appeared to be much smaller, confirming that it is becoming increasingly more frequent for COPD patients to die “with” rather than “due to” COPD.

This study displays a number of strengths. Firstly, it analyzes the overall data of a stand-alone health system covering a catchment population of approximately 2,700,000. This study involved no sampling, since it included the entire stratum of deceased COPD patients (with the ICD codes indicated) across the whole period analyzed and was thus fully representative. It should be noted that Galicia was the first Spanish autonomous region, and indeed one of the first regions in Europe, to have universal electronic health records for its entire healthcare system, with the possibility of accessing these to correct important incongruences or inconsistencies in death certificates, thus equipping it with one of the most advanced health information systems in Europe. When it comes to cause of death, the fact that the study targeted a single health service means that, even though there might conceivably be errors in the completion of death certificates, such errors would be expected to affect all the years recorded in the same way. Likewise, any variations would be expected to be fewer than if sampling had been performed or if different health services, possibly with a different diagnostic or registration criterion, had been included.

This study also has limitations. It is a descriptive study, and we have no data on factors that have an influence on COPD mortality, such as smoking habit, medication use, and patients’ previous admissions. Although this information might have been of great utility, it in no way detracts from the validity of the study, whose main aim was to ascertain the COPD mortality trend and the possible influence of multiple causes of death on the death register. A further limitation is that very few years—only three—were analyzed to assess the impact of multiple-cause coding. Even so, we feel that these provide sufficient evidence of how COPD mortality may be affected by a new system of analysis that includes multiple causes.

In conclusion, since 1996, COPD mortality has fallen steadily in Galicia in both sexes, without variations in the male:female mortality ratio over time. It is extremely positive that patients’ median age of death is practically equal to their life expectancy—something that reflects the effectiveness of these treatments and indirectly indicates that clinical practice is proving successful. Furthermore, the new multiple-mortality coding system may pose a real challenge to studies which seek to analyze COPD mortality, making it necessary to collect data not only on the underlying cause of death, but also on the immediate and intermediate cause or other processes if one wishes to avoid underestimating this particular cause of death.

## Figures and Tables

**Table 1:** Deaths and median age of death in the population of Galicia aged 35 years and over, due to COPD coded as underlying cause of death, both overall and in men and women. Galicia, population aged 35 years and over, period 1980–2017.

			Men	Women	Total
Number of deaths		1980–1989	5740	2609	8349
		1990–1999	9289	4028	13,317
		2000–2009	8823	3633	12,456
		2010–2017	6531	2581	9112
		Total	30,383	12,851	43,234
Age	Mean	1980–1989	76.0	79.6	77.1
		1990–1999	78.2	82.7	79.5
		2000–2009	80.7	85.5	82.1
		2010–2017	82.3	87.0	83.6
		Total	79.4	83.7	80.7
	25th percentile	1980–1989	71	75	72
		1990–1999	72	78	74
		2000–2009	76	81	77
		2010–2017	78	83	79
		Total	74	79	75
	50th percentile	1980–1989	77	81	78
		1990–1999	79	84	81
		2000–2009	82	87	83
		2010–2017	84	88	85
		Total	81	85	82
	75th percentile	1980–1989	82	85.5	84
		1990–1999	85	88	86
		2000–2009	87	91	88
		2010–2017	89	92	90
		Total	86	90	87

**Table 2:** Joinpoint analysis of the trend in standardized mortality rates due to COPD as underlying cause of death. Galicia, population aged 35 years and over, period 1980–2017. (APC: annual percentage change; CI: confidence interval).

Sex	Change Points (95% CI)	Period	APC (95% CI)		Interpretation
		1980–1985	11.5	(6.6; 16.6)	Rising
	1985 (1983; 1992)				
Men		1985–1996	2.0	(0.4; 3.6)	Rising
	1996 (1992; 2006)				
		1996–2017	−3.8	(−4.3; −3.3)	Falling
		1980–1996	2.1	(1.0; 3.2)	Rising
Women	1996 (1993; 1998)				
		1996–2017	−4.7	(−5.4; −4.1)	Falling
		1980–1989	6.0	(4.0; 8.0)	Rising
	1989 (1983; 1992)				
Total		1989–1996	0.5	(−3.0; 4.0)	Slightly rising
	1996 (1993; 2002)				
		1996–2017	−3.8	(−4.3; −3.3)	Falling

**Table 3:** Number of deaths in which COPD was shown as underlying cause and as immediate, intermediate, or other process, without being the underlying cause. Galicia, population aged 35 years and over, period 2015–2017.

Year	Deaths Due to COPD Considered as Underlying Cause	COPD Shown as Immediate or Intermediate Cause but Not as Underlying Cause	COPD Shown in Other Processes, but Not as Immediate, Intermediate, or Underlying Cause	Total
2015	1135	310	447	1892
2016	1064	327	429	1820
2017	1028	376	455	1859
Sum	3227	1013	1331	5571

## Data Availability

Data available under request.
